# Basic Clinical Bidirectional Empowerment: Synergistic Breakthrough in Molecular Mechanisms and Clinical Management of Small Cell Cervical Carcinoma

**DOI:** 10.3390/ijms27156783

**Published:** 2026-07-29

**Authors:** Mengjia Huang, Shuang Li

**Affiliations:** 1 Department of Obstetrics and Gynecology, National Clinical Research Center for Obstetrics and Gynecology, Tongji Hospital, Tongji Medical College, Huazhong University of Science and Technology, Wuhan 430000, China; m202476872@hust.edu.cn; 2 Key Laboratory of Cancer Invasion and Metastasis (Ministry of Education), Hubei Key Laboratory of Tumor Invasion and Metastasis, Tongji Hospital, Tongji Medical College, Huazhong University of Science and Technology, Wuhan 430000, China

**Keywords:** SCCC, hr-HPV, molecular mechanisms, basic clinical bidirectional empowerment

## Abstract

Small cell carcinoma of the cervix (SCCC) is a rare yet highly aggressive subtype of cervical cancer (CC), characterized by early invasion, high metastatic potential, frequent recurrence, and extremely poor prognosis, which severely impairs women’s physical and mental health. Currently, high-risk human papillomavirus (hr-HPV, particularly HPV18) infection is recognized as one of the core drivers underlying the initiation and progression of SCCC. Malignant evolution is not triggered by a single infection event but is cooperatively regulated at multiple molecular levels, including HPV genome integration, critical gene mutations, and aberrant activation of multiple signaling pathways. In addition, SCCC exhibits an HPV-independent oncogenic pathway mainly mediated by somatic mutations in tumor protein 53 (*TP53*) and retinoblastoma 1 (*RB1*), thus forming a dual pathogenic mechanism. Based on current clinical understanding and molecular mechanisms, this paper reviews the molecular pathogenesis and subtypes of SCCC, reveals the associations between tumor heterogeneity, therapeutic resistance, and metastasis, and proposes potential novel targets for the treatment of SCCC. This study innovatively presents a closed-loop model of two-way empowerment between basic research and clinical application. It emphasizes that basic research should be oriented toward real clinical problems and highlights the reciprocal feedback of clinical practice on basic research, thereby achieving dynamic iteration and collaborative breakthroughs in both fields.

## 1. Introduction

Globally, cervical cancer (CC) ranks as the fourth most common cancer in women, after breast, colorectal, and lung cancers, and represents a major threat to female health and survival. Small cell carcinoma of the cervix (SCCC) represents a rare histopathological subtype of cervical cancer, accounting for only 1–2% of all cervical malignancies [1,2]. It was first described by Reagan et al. in 1957 and has been referred to by various designations [3], including small cell neuroendocrine tumor and undifferentiated carcinoma [4,5]. It was not until the publication of the 2007 General Principles for Clinical and Pathological Management of the Uterine Cervix that SCCC was formally recognized as a distinct entity, based on its unique and aggressive biological behavior [6,7,8]. The epidemiological profile of SCCC exhibits marked distinctiveness. It is characterized by high invasiveness and an exceptionally aggressive biological course, making it one of the most lethal forms of cervical cancer. Tumor cells can breach cervical tissue boundaries early on, infiltrate surrounding structures, metastasize to lymph nodes, and hematogenously disseminate to distant organs such as the lungs, liver, bones, and brain [2,9,10,11,12]. This biological propensity directly translates into a profoundly poor clinical prognosis. Even among patients diagnosed at early stages, the recurrence rate exceeds 50% within two years. Across all stages, the overall five-year survival rate is dismal, ranging from approximately 13% to 25% [2,9,13], which is significantly lower than that of cervical squamous cell carcinoma (CSCC) or cervical adenocarcinoma (CA) [12,14,15,16,17].

Current studies have not identified significant racial specificity in SCCC; however, some data suggest that the incidence may be slightly higher among African American women compared to other racial groups [18]. This may be attributed to a higher prevalence of high-risk human papillomavirus (hr-HPV) infection and relatively limited healthcare access within this population. The age at diagnosis of SCCC spans a broad range, with the majority of cases occurring in women aged 40–60 years, although patients in their 20s and 30s have also been reported [18]. This differs somewhat from the peak incidence age of 45–55 years for squamous cell carcinoma, suggesting that younger women may be at a relatively higher risk for SCCC. Pregnancy-associated SCCC is extremely rare, with only a few cases documented in the literature [19,20,21,22,23]. The mortality rate of SCCC is significantly higher than that of common cervical cancer histological subtypes, with reported 5-year mortality rates as high as 60–80%. This far exceeds the rates for squamous cell carcinoma (<30%) and adenocarcinoma (~40%). Smoking, which may lead to immunosuppression, is a recognized risk factor for SCCC [24,25,26,27]. It increases the risk by approximately 2- to 3-fold, potentially by altering the local cervical microenvironment. Furthermore, harmful substances in tobacco can directly damage cervical cell DNA, which may act synergistically with HPV in carcinogenesis. High-risk human papillomavirus infection (particularly with types 16 and 18, and especially HPV18) is a central etiological factor in SCCC, with 70–90% of cases testing positive for the virus. Immunosuppressed individuals exhibit a reduced capacity to clear HPV infections, facilitating viral persistence and subsequent malignant transformation of cervical cells. A family history of cervical cancer, early age at first sexual intercourse, multiple sexual partners, high parity, long-term use of oral contraceptives, and co-infection with herpes simplex virus type 2 or human immunodeficiency virus are also factors that may increase the likelihood of persistent HPV infection and promote the malignant transformation of cervical cells [24].

From the perspective of bridging basic research and clinical practice, two core dichotomies persist. On one hand, the molecular pathogenesis of SCCC has not been fully elucidated, and key aspects such as the interaction between HPV infection and host genes as well as dysregulation of signaling pathways remain insufficiently explored. On the other hand, clinical management faces multiple challenges, including insufficient diagnostic specificity, a lack of targeted therapeutic regimens, and pronounced therapeutic resistance. As a result, most patients are diagnosed at locally advanced or metastatic stages, with only a limited subset of those with localized disease eligible for curative-intent treatment. Therefore, a critical pathway to overcome the bottlenecks in SCCC diagnosis and therapy lies in starting from its molecular foundations, deducing the mechanisms underlying its clinical phenotypes, and translating these insights into clinical strategies for diagnosis, treatment, and prognosis management. This approach would not only provide patients with more treatment opportunities and time but also offer a stronger safeguard for women’s health.

## 2. Traditional Clinical Understanding of SCCC

### 2.1. Clinical Diagnosis and Treatment System and Its Limitations

#### 2.1.1. Pathological Diagnostic Criteria and Application of Immunohistochemical Markers in SCCC

Histopathological examination reveals that primary small cell carcinoma of the cervix displays morphological features similar to those of pulmonary small cell carcinoma. Their shared pathological features include densely packed small tumor cells with a predominantly diffuse growth pattern [28], with tumor cells arranged in nests, sheets, trabeculae, cords, and rosette-like formations; peripheral areas may show palisading or protrusions [29,30]. The tumor is often friable and commonly associated with extensive geographic necrosis [28]. Microscopically, small round or spindle-shaped cells with uniform size are observed, featuring scant basophilic cytoplasm, hyperchromatic nuclei, inconspicuous nucleoli, numerous mitotic figures, and frequent necrosis and apoptotic bodies [29,31]. The chromatin is fine, granular in appearance [29]. SCCC is classified into pure and mixed types, with the mixed type frequently associated with squamous cell carcinoma or adenocarcinoma, though the proportion of the small cell component varies [29]. Following treatment, tumor cells may show morphological variations, such as cellular enlargement and prominent nucleoli.

Immunohistochemistry (IHC) is helpful in confirming neuroendocrine differentiation, but it is not mandatory for the diagnosis of SCCC if histologic criteria are fulfilled [31,32]. The evolution of IHC consensus reflects the refinement of clinical standards by basic research. Commonly used IHC markers for SCCC include chromogranin A (CgA), synaptophysin (Syn), neural cell adhesion molecule (CD56), and neuron-specific enolase (NSE); their positive expression suggests a tumor origin from the neuroendocrine system [33,34,35,36]. Small cell lung cancer (SCLC) and SCCC are both classified as high-grade neuroendocrine malignancies. Both consistently express the core neuroendocrine markers CD56, Syn, CgA and NSE, which forms the pathological basis for extrapolating therapeutic regimens from SCLC to SCCC in clinical practice [37,38,39]. In clinical practice, many studies have emphasized that dual or multiple marker expression is a necessary diagnostic criterion, yet due to variable positivity rates and unclear prognostic significance, diagnostic challenges persist [40,41]. The differences in expression levels of the four neuroendocrine markers are related to their molecular characteristics: Syn is a calcium-binding protein located on synaptic vesicle membranes and is diffusely expressed in the cytoplasm of neuroendocrine cells [41]; its molecular biological properties may explain its high expression. CD56 expression is second only to Syn and shows less heterogeneity than the other three markers; as a cell surface glycoprotein and a member of the cell adhesion molecule family [42], CD56 plays an important role in tumor cell invasion and metastasis. Its high expression aligns with the aggressive nature of SCCC, and it exhibits stable expression when detected by IHC methods. Among the markers NSE and CgA, CgA in particular shows a relatively lower positive expression rate [33]. McCluggage et al. [43] found that Syn and CD56 are the most sensitive neuroendocrine IHC markers [33], while NSE has high sensitivity but lower specificity, and CgA has the strongest specificity. Insulinoma-associated protein 1 (INSM1) and secretagogin (SCGN) are promising diagnostic markers with high sensitivity and specificity and can also be used to demonstrate neuroendocrine differentiation [44,45,46].

#### 2.1.2. Practical Value and Limitations of Routine Screening Methods

The development of diagnostic techniques continues to revolve around the molecular characteristics of SCCC and is constantly optimized in clinical application. HPV infection can be detected by various methods (polymerase chain reaction [PCR], IHC, and in situ hybridization [ISH]) [12,47]. The high sensitivity of HPV detection is related to the strong association between HPV16 and HPV18 and SCCC confirmed by basic research. Although HPV is present in almost all cervical squamous cell carcinomas and adenocarcinomas, it is detected in only about 50% of SCCC cases [48,49], yet HPV testing is more sensitive than cytology. Liquid-based cytology is a classic method for cervical cancer screening, allowing observation of cellular morphology for features of precancerous lesions or cancer cells, but it has a relatively high rate of missed diagnosis and misdiagnosis for SCCC [36,50]. This may be related to the difficulty in recognizing the characteristic “salt-and-pepper” chromatin pattern of SCCC under the microscope.

When HPV testing or cytology results are abnormal, further pathological analysis is required. Colposcopy-guided biopsy serves as the “gold standard” for definitive diagnosis. Its pathological analysis is based on the unique histological features of SCCC, which are associated with HPV infection-driven cell cycle dysregulation, abnormal activation of oncogenes, and inactivation of tumor suppressor genes. Immunohistochemical examination further provides molecular validation of the tumor’s neuroendocrine differentiation by detecting neuroendocrine markers such as Syn, CgA, NSE, and CD56 [33,34,35,36].

#### 2.1.3. Optimization of the Staging System for SCCC and the Applied Value of Imaging Evaluation

Imaging examinations are also indispensable in assessing disease progression. Ultrasound can preliminarily determine the size of cervical masses and pelvic involvement, but its sensitivity for detecting microscopic metastases is limited. MRI, recommended by the European Society of Urogenital Radiology guidelines as a useful method for initial staging, treatment monitoring, and recurrence assessment of cervical cancer due to its excellent soft-tissue resolution and non-invasive nature [51], offers superior soft-tissue contrast. CT is more advantageous in detecting distant metastases but has lower soft-tissue resolution compared to MRI. Positron emission tomography–computed tomography PET-CT demonstrates high sensitivity and specificity in detecting recurrent tumors [52,53,54,55].

Staging of small cell carcinoma of the cervix (SCCC) currently primarily follows the FIGO 2018 staging system, which incorporates imaging findings (e.g., CT, PET/CT) and pathological results for tumor staging [2,56]. This system, based on the extent of tumor invasion and metastasis, provides crucial guidance for treatment selection and prognosis evaluation. In low-income regions, the accuracy of staging may be affected by limited availability of imaging equipment. Compared to FIGO 2009, the pathologically based FIGO 2018 staging is more accurate regarding pathological parameters such as vaginal involvement, parametrial involvement, tumor size, lymph node metastasis, depth of tumor invasion, and lymphovascular space invasion (LVSI). A major change in FIGO 2018 is the subdivision of stage IB from two subgroups into three subgroups based on tumor diameter (≤4 cm and >4 cm vs. ≤2 cm, 2–4 cm, and >4 cm), aimed at identifying patients with tumors ≤ 2 cm who may be candidates for fertility-sparing surgery. Another significant change is the introduction of stage IIIC based on lymph node metastasis (LNM) to improve prognostic prediction. The FIGO 2018 staging system, when combined with the degree and sites of local tumor invasion and lymph node metastasis, may provide a more individualized and accurate assessment. For stage II SCCC patients, FIGO 2009 tended to underestimate prognosis, which could negatively impact patient psychology and hinder precise treatment. In contrast, FIGO 2018 includes two intermediate-risk factors—depth of tumor invasion and LVSI—thereby enabling a more accurate prediction of patient prognosis.

### 2.2. Clinical Treatment Strategies and Therapeutic Challenges in SCCC

Cervical HPV infection is considered to occur at some point in the lifetime of most women, being most prevalent after the initiation of sexual activity. However, approximately 90% of individuals clear the HPV infection within two years due to their own immunity, yet in a substantial proportion of individuals, the infection persists. Preventive vaccination can prevent cervical infection [57], but it is ineffective for women who have already established an HPV infection.

Current National Comprehensive Cancer Network (NCCN) guidelines for cervical cancer explicitly exclude small cell histology, only indicating that it is unsuitable for fertility-sparing surgery, and provide almost no guidance for the treatment of this disease [58,59]. Some scholars believe that SCCC shares aggressive similarities with SCLC [59,60], hence the treatment paradigm is analogous [61,62,63,64,65]. Nevertheless, the two tumor types exhibit fundamental heterogeneity in terms of pathogenic drivers, genomic features, and tumor immune microenvironment [38,66]. SCLC is predominantly driven by long-term tobacco exposure; tobacco-related carcinogens trigger genomic damage, and intrinsic somatic mutations of *TP53* and *RB1* represent its core oncogenic events [37,67]. In contrast, the vast majority of SCCC originates from persistent high-risk HPV infection. It primarily utilizes E6/E7 oncoproteins to mediate degradation of tumor suppressor proteins and perturb cell cycle control. Accordingly, the two tumors display marked differences in core oncogenic pathways and global mutational landscapes [37,67]. Meanwhile, the two malignancies exhibit different immune microenvironment phenotypes, and their characteristics of response to chemotherapy and immunotherapy together with drug resistance patterns are not fully consistent [68]. Accordingly, treatment approaches for SCLC cannot be completely extrapolated to SCCC, and individualized diagnosis and treatment strategies based on its distinct molecular features are urgently required [37,67,69].

Given the aggressiveness of this disease, the management strategies for small cell carcinoma of the cervix issued by the Society of Gynecologic Oncology (SGO) and the Gynecologic Cancer InterGroup (GCIG) recommend a multimodal treatment approach [2,12]. For early-stage patients without bulky tumors, the guidelines recommend radical hysterectomy with lymphadenectomy, with or without salpingo-oophorectomy, followed typically by adjuvant chemotherapy with cisplatin and etoposide ± radiotherapy [70,71]. This regimen is designed based on foundational research confirming that surgical removal of local lesions can reduce sources of metastasis, chemotherapy can control distant metastasis, and radiotherapy controls local disease [72]. For early-stage but bulky tumors > 4 cm, a strategy of neoadjuvant chemotherapy to shrink the lesion first followed by surgery is considered, based on the molecular mechanism that chemotherapy can inhibit tumor proliferation and downstage the disease. Although this can improve the resectability rate in locally advanced patients, its clinical application remains controversial and requires further evaluation of efficacy combined with molecular biomarkers [2,73]. Locally advanced patients are treated with chemoradiotherapy followed by systemic chemotherapy, and patients with distant metastasis receive palliative care [74].

Furthermore, while managing complications with early detection and treatment, supportive care should also be considered, including granulocyte colony-stimulating factors to maintain the treatment schedule and prevent neutropenia-related infections. During follow-up, psychological support should be provided to patients, and personalized rehabilitation plans should be developed according to the specific situation of the patient to improve quality of life. These clinical decisions correspond to the molecular characteristics of SCCC where radiotherapy controls local disease and chemotherapy controls systemic disease. Although multimodal treatment can improve disease-free survival (DFS) [75], its prognosis remains worse than other cervical cancer subtypes, highlighting the urgent clinical need for novel therapies [74].

## 3. Molecular Mechanisms: The Foundational Logic Underlying the Malignant Phenotype of SCCC

In the initial phase of clinical research on SCCC, significant limitations in diagnostic and therapeutic cognition restricted the identification of human papillomavirus (HPV) infection predominantly to HPV16/18, with HPV18 being the most prevalent subtype. SCCC is characterized by core features of exceptionally high malignancy, rapid progression, swift metastasis, and extremely poor prognosis. However, the fundamental nature of its pathogenesis and the underlying causes of its aggressive and metastatic behavior remained poorly understood. The clinical phenotype of SCCC is highly insidious, with precursor lesions being nearly undetectable by conventional means, leading to a high rate of missed early diagnoses. Consequently, most patients were diagnosed at an advanced cancer stage, making it clinically challenging to implement timely interventions for effective disease control.

To address this critical bottleneck, our research focus has shifted toward investigating the molecular characteristics of SCCC. By conducting in-depth explorations of its molecular mechanisms, we aim to unravel core molecular events, including the interaction patterns between HPV infection and host genes, genomic integration modes, aberrant signaling pathways and the effects of epigenetic regulation. These investigations will elucidate the biological essence underlying SCCC’s high malignancy and metastatic propensity, thereby providing theoretical support for overcoming current limitations in clinical diagnosis and treatment.

### 3.1. HPV Infection and Oncogenic Interactions with Host Cells: The Initial Driver of Tumorigenesis

Small cell carcinoma of the cervix (SCCC) is closely associated with high-risk human papillomavirus (hr-HPV) infection, although HPV is not universally detected in all SCCC specimens [76]. HPV is a small, non-enveloped, double-stranded circular DNA virus, approximately 50–60 nm in diameter and about 8000 base pairs in length, with an icosahedral capsid symmetry. Its genome is divided into the E region, the L region, and the long control region (LCR) or upstream regulatory region (URR): the E region plays a key role in viral replication, transcriptional regulation, and oncogenic transformation; the L region is involved in virion assembly and viral structure; and the LCR contains the origin of replication as well as promoters and enhancers for transcription. As a double-stranded DNA virus, high-risk HPV infection exhibits cell specificity, occurring predominantly in epithelial cells, with very low infection rates in stromal and immune cells [77,78]. Hr-HPV infection is a central etiological factor in SCCC, with HPV18 being the predominant subtype [79,80,81], followed by HPV16 and HPV35, while other subtypes are rare [37,81,82,83,84,85]. HPV18 shows a strong correlation with neuroendocrine differentiation features, whereas HPV16 is more frequently associated with squamous differentiation. Moreover, compared to cervical squamous cell carcinoma and adenocarcinoma, SCCC tends to harbor a single HPV subtype infection, which represents a significant difference from other cervical cancer subtypes.

The early region E proteins of human papillomavirus (HPV) constitute the core viral coding elements, among which E1 and E2 are the primary functional proteins activated after HPV invades host cells. As a viral DNA helicase, E1 initiates viral genome replication; E2 is capable of specifically binding to DNA lesion sites and physically interacting with TopBP1, a master host protein involved in DNA damage repair [86,87]. TopBP1 forms a ternary complex with E1 and E2 to ensure smooth and ordered viral genome replication. This protein harbors nine structural domains at its C-terminus that fold into hydrophobic pockets, which are capable of binding damaged DNA, phosphorylated proteins, and diverse host functional molecules [86,88]. In addition, E2 preserves the episomal state of viral genomes by binding to the viral origin of replication, and exerts transcriptional repression on the E6/E7 promoters. Thus, E2 acts as a critical “housekeeping factor” during HPV persistent infection [86,89].

**Figure 1:**
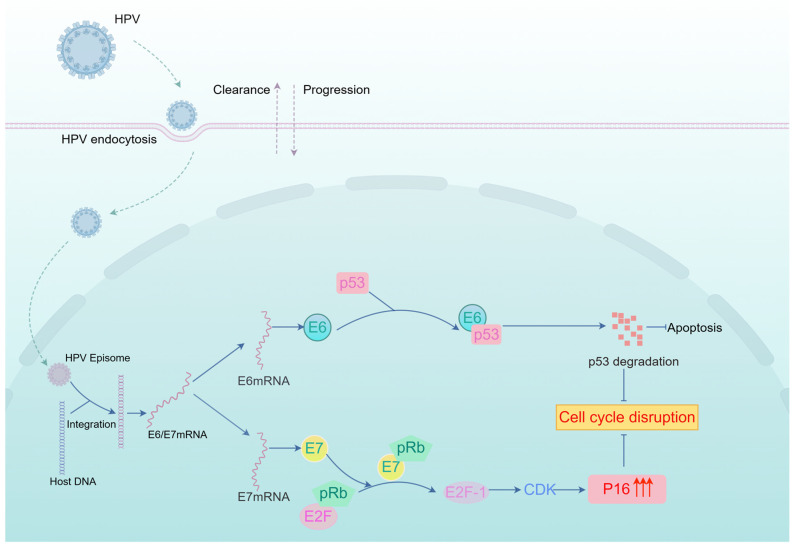
Carcinogenic interaction between human papillomavirus (HPV) infection and host cells: regulation of cellular immortalization by E6/E7 via the p53/retinoblastoma protein (pRb) pathway. In the figure, light green dashed arrows indicate biological trafficking, intracellular transport and event progression. The upward purple dashed arrow stands for host clearance of HPV infection, and the downward purple dashed arrow indicates persistent HPV infection that fails to be cleared. Triple red upward arrows signify markedly elevated protein levels, and T-shaped arrows represent inhibition. The figure was made by Figdraw (www.figdraw.com).

As the infection progresses, the HPV genome integrates into host chromatin, which disrupts the E2 gene and abolishes its expression. Since E2 negatively regulates the transcription of E6 and E7, loss of E2 is accompanied by the deletion or transcriptional silencing of the E1 gene simultaneously [90]. As a potential tumor suppressor, loss of E2 relieves transcriptional repression of the E6 and E7 promoters, thereby driving robust overexpression of E6 and E7 oncoproteins [90]. The E6 oncoprotein binds to and degrades wild-type p53 via the ubiquitin–proteasome pathway, inactivating its function while simultaneously activating telomerase to maintain cellular immortality, thereby providing a basis for unlimited proliferation of tumor cells [48,49,91,92,93]; the E7 protein binds to and inactivates the retinoblastoma protein (pRb) tumor suppressor gene product, resulting in the release of the E2F-1 transcription factor from the E2F-pRb complex, which activates cyclin-dependent kinases (CDKs) and transcriptional activation of target promoters, subsequently triggering p16 overexpression through a feedback mechanism [94], thereby disrupting cell cycle regulation. Together, these two proteins coordinately dismantle cell cycle control, driving cellular immortalization and aberrant proliferation (Figure 1) [95].

This interaction is not merely an incidental phenomenon of viral infection; chronic inflammation also plays a non-negligible role in this process. Persistent infection with high-risk HPV induces a chronic low-grade inflammatory microenvironment in the cervix [96,97,98]. This microenvironment not only impairs local antiviral defense and viral clearance, but also exacerbates DNA damage and genomic instability via proinflammatory cytokines and oxidative stress. Such events constitute a vicious cycle that sustains persistent HPV infection and facilitates the progression of epithelial lesions [96,97,98]. Moreover, in situ hybridization detection of HPV messenger ribonucleic acid (mRNA) confirms active viral transcription within tumors [82], and HPV infection exhibits a synergistic effect with SRY-box transcription factor 2 (SOX2) overexpression [99], jointly propelling cervical cells toward a malignant phenotype. Unlike the oncogenic mode in HPV-positive SCCC, HPV-negative SCCC frequently relies on somatic mutations in *TP53* (50%) and retinoblastoma 1 (*RB1*) (75%) [100], suggesting that SCCC encompasses both HPV-dependent and HPV-independent molecular pathogenic pathways. This finding provides a foundational basis for subsequent clinical stratification and treatment.

### 3.2. HPV Genomic Integration Patterns: A Stepwise Reinforcement of Malignant Phenotype

**Figure 2:**
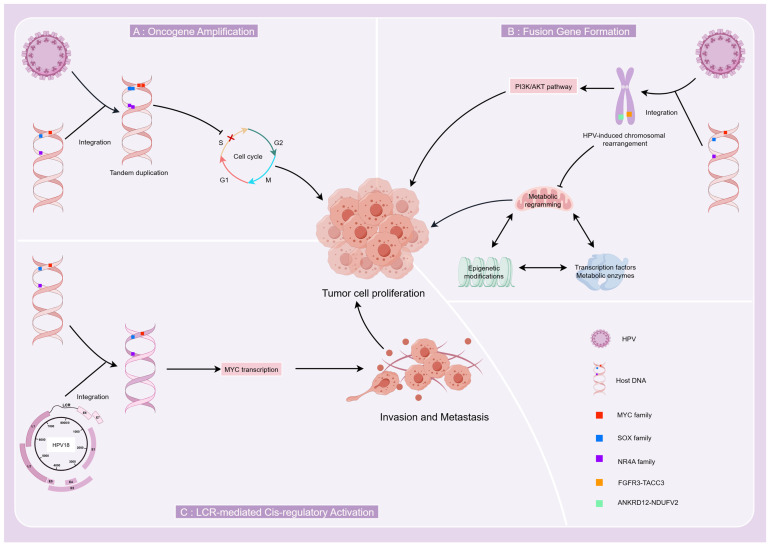
Three major modes of human papillomavirus (HPV) integration-driven tumor progression. (**A**) Oncogene amplification: HPV integrates into host family genes, inducing tandem duplication of host genomic fragments. This leads to increased copy number and overexpression of oncogenes, disrupts cell cycle homeostasis, and ultimately promotes malignant proliferation of cells; (**B**) Fusion gene formation: HPV integration induces structural variations in host chromosomes. It further generates two fusion genes: fibroblast growth factor receptor 3-transforming acidic coiled-coil containing protein 3 (FGFR3-TACC3), which activates the PI3K/Akt signaling pathway, and ankyrin repeat domain-containing protein 12-NADH dehydrogenase flavoprotein 2 (ANKRD12-NDUFV2), which disturbs mitochondrial function and enhances metabolic reprogramming. Collectively, these two fusion genes drive malignant cell proliferation; (**C**) LCR-mediated cis-regulatory activation: integration of the HPV18 LCR inserts within 500 kb upstream of the MYC proto-oncogene family (MYC) gene. It enhances the transcriptional efficiency of MYC via cis-regulation, thereby increasing tumor invasiveness and driving malignant cell proliferation. In the figure, double-headed black arrows represent reciprocal interaction and mutual regulation. T-shaped arrows indicate inhibition or blockade, and regular single-headed black arrows signify promotion or activation. The figure was made by Figdraw (www.figdraw.com).

Britta Weigelt and colleagues found that virus-negative tumors require multiple genetic events to induce malignant transformation, whereas viral integration acts as a strong oncogenic event in virus-positive tumors [101]. Integration of the HPV genome into host chromosomes is a key molecular event driving the increased malignancy of SCCC. Research by Wang et al. identified, in addition to the MYC proto-oncogene family (MYC) genes (37.9%), the SRY-box transcription factor family (SOX) genes (8.4%), nuclear receptor subfamily 4 group A (NR4A) genes (6.3%), ankyrin repeat domain-containing family (ANKRD) genes (7.4%), and carcinoembryonic antigen (CEA) (3.2%) genes as hot spots for HPV integration. They found that HPV18 tends to integrate into the MYC family, while HPV16 more frequently integrates into the SOX2 and NR4A families. Nearly half of the samples exhibited dual oncogene integration, and this integration pattern drives tumor progression through three co-existing integration modes (Figure 2): oncogene duplication, fusion gene formation, and cis-regulatory activation of genes via the viral LCR (MYC), with tandem duplication and LCR amplification being key mechanisms [102]. In the oncogene duplication mode, HPV integrates into regions of oncogene families such as MYC (MYC, MYCN, MYCL, accounting for 37.9%) and SOX (SOX2 and its paralogs, 8.4%), triggering tandem duplication of host genomic segments, which leads to copy-number gain and overexpression of these oncogenes [102]. Among these, the MYC family, as a core oncogene regulating cell proliferation and apoptosis, disrupts cell-cycle homeostasis when overexpressed and thereby promotes malignant cell proliferation. The SOX family, NR4A family (6.3%), and others can similarly enhance their aberrant regulatory functions through copy-number amplification (Figure 2A). In the fusion-gene formation mode, HPV integration induces structural variations in host chromosomes, generating fusion genes such as fibroblast growth factor receptor 3-transforming acidic coiled-coil containing protein 3 (FGFR3-TACC3) and ankyrin repeat domain-containing protein 12-NADH dehydrogenase flavoprotein 2 (ANKRD12-NDUFV2) [102]. FGFR3-TACC3 activates the PI3K/Akt signaling pathway to promote cell survival and proliferation, while ANKRD12-NDUFV2 may interfere with mitochondrial function to enhance metabolic reprogramming, together reinforcing the malignant phenotype of the tumor (Figure 2B). In the cis-regulatory activation mode, the HPV18 LCR, rich in transcription-factor binding sites and epithelial-specific viral enhancers, integrates within 500 kb upstream of MYC and significantly enhances MYC transcriptional efficiency through cis-regulation, thereby increasing tumor invasiveness and driving malignant cell proliferation (Figure 2C) [102]. This activation mode, independent of oncogene copy-number increase, has been demonstrated in HeLa cells to markedly elevate tumor aggressiveness [103].

### 3.3. Endogenous Molecular Events: The Cornerstone for Maintaining the Malignant Phenotype

**Figure 3:**
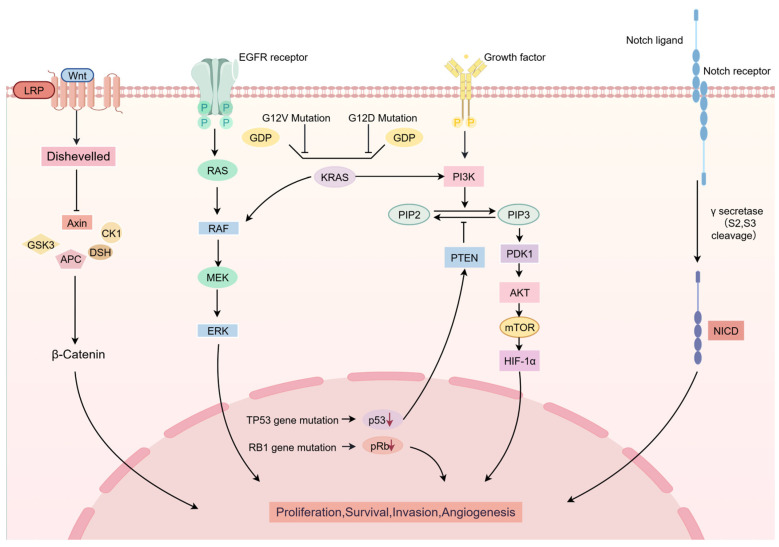
Molecular networks composed of key gene mutations and signaling pathway abnormalities in small cell cervical cancer. In the figure, red downward arrows indicate reduced protein abundance or protein inactivation. T-shaped arrows represent inhibition, and regular arrows signify promotion or activation. The figure was made by Figdraw (www.figdraw.com).

Compared with conventional cervical cancer, SCCC exhibits a low tumor mutational burden and fewer copy number alterations [101], placing it at an intermediate level among all tumors in The Cancer Genome Atlas (TCGA) [68]. However, key gene mutations and signaling pathway abnormalities collectively form a molecular network that sustains the malignant phenotype (Figure 3).

#### 3.3.1. Key Gene Mutations and Genomic Instability

Key genetic mutations and copy number variations (CNVs) contribute to the pathogenesis of small cell carcinoma of the cervix (SCCC) by disrupting cell signaling pathways, regulating cell proliferation, and impairing genomic stability. Frumovitz et al. identified that at least one targetable mutation exists in patients with SCCC [76]. The three most frequently mutated genes across all SCCC cases are phosphatidylinositol-4,5-bisphosphate 3-kinase catalytic subunit alpha (PIK3CA), *TP53*, and KRAS proto-oncogene, GTPase (KRAS) [67,76,104,105,106]. Mutations in PIK3CA, phosphatase and tensin homolog (PTEN), and other related genes aberrantly activate the PI3K/Akt/mTOR pathway. Sustained activation of this pathway promotes cell survival, proliferation, and migration, endowing tumor cells with a growth advantage. Consequently, some cases exhibit sensitivity to mTOR inhibitors. The two most common mutations in KRAS are G12D and G12V (Figure 3): the G12D variant transmits signals primarily through pathways such as PI3K, focal adhesion kinase (FAK), and c-Jun N-terminal kinase (JNK), with minimal reliance on the rapidly accelerated fibrosarcoma (RAF)/extracellular regulated protein kinase (ERK) pathway [107,108], whereas the G12V variant relies on mitogen-activated protein kinase (MAPK) cascade signaling and loses the ability to bind and signal through PI3K, particularly by blocking protein Kinase B (Akt) activation [107]. Dysregulation of this pathway drives the transmission of cell proliferation signals. These differences between the two mutation types provide a basis for targeted drug selection: tumor cells harboring the G12V mutation may be more sensitive to selective mitogen-activated protein kinase kinase (MEK) inhibitors, whereas those with the G12D mutation may require combined inhibition of both the MAPK and PI3K pathways. Unlike HPV-positive SCCC, HPV-negative SCCC exhibits a significantly higher mutation rate of *TP53* and a high incidence of *RB1* mutations. Loss-of-function mutations in these genes abrogate their tumor suppressor activities, suggesting the existence of HPV-independent oncogenic pathways that are closely associated with the high invasiveness of the tumor [109]. Beyond these common driver mutations, genes such as erb-b2 receptor tyrosine kinase 4 (ERBB4), alpha thalassemia/mental retardation syndrome X-linked (ATRX), and fibroblast growth factor receptor-like 1 (FGFRL1) may also facilitate tumorigenesis through dysregulation of their respective cellular functions [104], failing to effectively suppress malignant transformation.

Focal amplifications are frequently observed in SCCC, including regions 3q26.3-q27 and oncogenes such as the SOX2 and MYC families, which lead to increased expression of the corresponding genes. SOX2 is highly expressed due to its localization within an amplified genomic region [99], and this aberrant expression synergizes with high-risk HPV infection to accelerate the malignant transformation of tumor cells. Amplification of MYC family genes further promotes carcinogenesis and progression by enhancing their regulatory effects on cell proliferation and apoptosis.

#### 3.3.2. Abnormal Activation of Core Signaling Pathways

Aberrant activation or dysregulation of multiple key signaling pathways plays a crucial driving role in the tumorigenesis and progression of SCCC (Figure 3). Genetic alterations involving pathways such as PI3K/Akt/mTOR and MAPK have been identified in SCCC [104], with aberrations in the PI3K/Akt/mTOR pathway being the most prevalent. As a core regulatory axis governing cellular metabolism, survival, and invasive capacity, the PI3K/Akt/mTOR pathway is frequently activated and functionally disrupted by molecular events including PIK3CA gene mutations, PTEN inactivation, and RICTOR mutations. This dysregulation significantly enhances the survival advantage of tumor cells, promotes abnormal proliferation and metastasis, and serves as a central target for current targeted therapeutic strategies [95]. Mutations in KRAS, ERBB2, and serine/threonine kinase 39 (STK39) lead to aberrant activation of the MAPK pathway, which drives the regulation of cell proliferation and differentiation, remarkably augmenting tumor invasive potential. Consequently, some cases harboring these mutations exhibit sensitivity to MEK inhibitors [95]. In addition, the aberrant activation of pathways such as hypoxia-inducible factor-1α (HIF-1α), Wnt/β-catenin and Notch/Hedgehog also plays critical roles in tumorigenesis (Figure 3) [76,102,110,111].

### 3.4. Epigenetic Abnormalities: Multilayer Regulatory Mechanisms Underlying Tumor Progression

Studies on epigenetics in SCCC remain in an early stage. Most regulatory hypotheses lack dedicated in vitro and in vivo experimental evidence, and further investigations are required to delineate its regulatory network. At the level of methylation regulation, the p16/pRb pathway exhibits a bidirectional regulatory pattern in SCCC. HPV E7-mediated degradation of pRb triggers compensatory overexpression of p16 [112], whereas CpG island hypermethylation of the p16 promoter silences p16 transcription and induces cell cycle disturbance [113]. Referring to evidence from cervical squamous cell carcinoma, downregulation of multiple miRNAs can also be regulated by promoter methylation, forming a methylation–miRNA–pRb regulatory axis that relieves the constraints on tumor-suppressive pathways to sustain malignant phenotypes. Nevertheless, relevant functional validations remain insufficient in SCCC [114]. At the level of non-coding RNA regulation, multi-omics subtyping reveals that miRNA abnormalities facilitate tumor invasion across distinct neuroendocrine subtypes [68,115], and single-cell sequencing verifies that malignant epithelial subpopulations harbor characteristic non-coding RNA profiles accompanied by an immune-exhausted microenvironment [110]. Multiple miRNAs are markedly downregulated in advanced SCCC, and low expression of certain miRNAs indicates poor prognosis [115]. To date, available evidence for lncRNAs in SCCC only demonstrates HPV18 integration into the intronic regions of lncRNA genes; its transcriptional and functional effects remain unclear, and relevant regulatory patterns are largely inferred from SCLC studies [37,101]. In terms of histone modifications, HPV18 E7 not only degrades pRb, but also recruits HDACs to reduce H3/H4 acetylation and induce chromatin compaction. Meanwhile, EZH2 mediates the enrichment of H3K27me3. Together, these two events synergistically repress cellular senescence and drive neuroendocrine malignant differentiation, constituting a two-layer epigenetic regulatory network [37,116]. Nevertheless, direct experimental evidence and systematic histone modification profiles specific to SCCC are still lacking.

## 4. Advancement in Clinical Diagnosis and Treatment: The Bidirectional Cycle Between Molecular Characteristics and Clinical Practice

As a rare cervical malignancy, the diagnostic framework for SCCC is fundamentally a scientific process that evolves through iterative validation between molecular mechanisms and clinical phenotypes. Clinical phenotypes represent in vivo natural experiments of molecular mechanisms. By adopting an analytical paradigm that moves from clinical observation to mechanistic investigation and back to clinical feedback, a precision medicine logic for SCCC can be constructed.

### 4.1. Mapping and Tracing Molecular Mechanisms to Clinical Features

The symptoms of SCCC are nonspecific, yet each clinical manifestation reflects an underlying molecular regulatory logic. The bidirectional validation between clinical observation and basic research has unveiled the regulatory mechanisms behind these symptoms.

Common clinical symptoms such as irregular vaginal bleeding and cervical mass with easy bleeding may result from tumor invasion into cervical stromal vessels or branches of the uterine arteries, leading to ischemia, necrosis, and bleeding upon detachment. The root cause lies in HPV-driven cell cycle dysregulation and vascular invasive properties. HPV18 E6 protein degrades p53 [48,49,91,93], and the E7 protein releases inhibition at the G1 cell cycle checkpoint [94], together leading to a Ki-67 index exceeding 80% in SCCC cells, forming an immortalized proliferation phenotype. This rapid proliferation increases intratumoral pressure while activating hypoxia-inducible factor (HIF-1α), which upregulates vascular endothelial growth factor (VEGF) expression and promotes the formation of abundant but fragile neovascularization [117]. These newly formed vessels lack a smooth muscle layer and are structurally unstable, making them prone to rupture during cervical friction or tumor invasion, resulting in bleeding.

During advanced stages of SCCC, aberrant activation of the PI3K/Akt/mTOR pathway upregulates matrix metalloproteinases (matrix metalloproteinase-2 (MMP-2) matrix metalloproteinase-9 (MMP-9)) [118], enhancing tumor invasiveness [119]. Excessively proliferative cells breach the cervical stromal barrier, invading parametrial tissues and pelvic wall nerves, causing lower abdominal and pelvic pain. Compression of the bladder or rectum may lead to obstructive symptoms such as urinary frequency and constipation, with the specific symptoms potentially varying based on the tumor location within the cervical canal [83].

Regarding the discrepancy between the theoretical neuroendocrine characteristics and the actual clinical phenotype, basic research has confirmed that SCCC highly expresses neuroendocrine transcription factors such as achaete-scute family bHLH transcription factor 1 (ASCL1) and neuronal differentiation 1 (NEUROD1) [68], which should drive the synthesis of hormones and neurotransmitters such as somatostatin and serotonin, potentially causing hormone-related symptoms. However, the majority of SCCC patients do not exhibit clinical manifestations of hormonal abnormalities [120], a paradox that has prompted further research. This may be because the hormones and neurotransmitters secreted by the tumor are often biologically inactive or rapidly degraded, or the quantities produced are insufficient to cause clinical symptoms. Nevertheless, a minority of patients may present with paraneoplastic syndromes such as carcinoid syndrome, Cushing’s syndrome, hypoglycemia, or syndrome of inappropriate antidiuretic hormone secretion, which are neuroendocrine in nature [83,120,121,122].

### 4.2. Revolution in Molecular Subtype-Driven Therapy

#### 4.2.1. Subtyping Based on Neuroendocrine Phenotype and Transcription Factors: A New Dimension for Guiding Treatment Sensitivity

Through single-cell RNA sequencing, malignant epithelial cells of SCCC were classified into four molecular subtypes based on the expression of key transcription factors such as ASCL1, NEUROD1, POU class 2 homeobox 3 (POU2F3), and yes-associated protein 1 (YAP1), namely the “A-N-P(Y) subtypes,” corresponding to the four epithelial cell clusters α, β, γ, and δ. The first two are classified as neuroendocrine phenotypes, while the latter two are considered non-neuroendocrine types [123,124]. The A-type (ASCL1^+^), belonging to the α cluster, is associated with cell cycle regulation and neuroendocrine functions. It participates in both the “Tu P/A” pathway, where dedifferentiated P-type cells ultimately form some A-type malignant cells, and the “Tu A/N” pathway involving high neuroendocrine differentiation from A-type to N-type cells. Some subtypes within this group are poorly differentiated, show moderate sensitivity to chemotherapy, and are prone to early recurrence. The N-type (NEUROD1^+^), belonging to the β cluster, highly expresses schlafen family member 11 (SLFN11) and mTOR. Basic research has confirmed that this subtype may benefit from PARP/mTOR inhibitors. The P-type (POU2F3^+^), belonging to the γ cluster, is related to keratinocyte evolution and exhibits the lowest degree of differentiation [123]. It lacks typical neuroendocrine features, enriches cluster of differentiation 8 (CD8^+^) exhausted T cells and secreted phosphoprotein 1 (SPP1^+^) macrophages, and presents a “hot tumor” phenotype. In contrast, A-type and N-type tumors exhibit a “cold tumor” phenotype [123,124,125]. Basic research shows a good response to immune checkpoint inhibitors such as platelet-derived growth factor (PDGF) receptor inhibitors in P-type tumors, making them a priority for considering immunotherapy in clinical practice. YAP1 is highly expressed mainly in normal epithelial cells, and its decreased expression in malignant cells suggests a poorer prognosis. Basic experiments have shown that histone deacetylase (HDAC) inhibitors can reverse its malignant phenotype, providing a therapeutic approach for clinical salvage therapy [123].

#### 4.2.2. Subtyping Based on Mutational Signatures: A Blueprint Linking Prognosis and Therapeutic Decision-Making

Analysis of tumor tissue using whole-exome sequencing or similar methods to identify mutational signatures and common mutated genes classifies SCCC into deficient mismatch repair (dMMR)-like and high APOBEC-mediated deamination subtypes. The dMMR-like subtype accounts for 40% of cases and is characterized primarily by dysfunction of the mismatch repair system, resulting in a high tumor mutational burden and enrichment of mutations in PI3K/Akt signaling pathway-related genes. The dMMR-like subtype exhibits weak DNA damage repair capacity, is associated with a higher clinical tendency for metastasis and advanced FIGO stage, and may be more aggressive, suggesting potential responsiveness to immune checkpoint inhibitors [126]. As this subtype likely involves oncogenic mechanisms related to PI3K/Akt pathway abnormalities, it also provides a rationale for targeted therapy [126], and patients could be prioritized for enrollment in immunotherapy clinical trials. The high APOBEC-mediated deamination subtype accounts for 60% of cases, with spontaneous deamination mutations being the primary mutational feature, mainly involving genes in the RAS signaling pathway. Compared to the dMMR-like subtype, this subtype is associated with earlier FIGO stage and a relatively lower metastasis rate. Therapeutically, exploration of MEK inhibitors or RAS inhibitor combinations is warranted.

#### 4.2.3. Subtypes Based on the Immune Microenvironment: Laying the Foundation for Precision Immunotherapy

Using multiplex immunofluorescence to detect the expression of cluster of differentiation 74 (CD74), ASCL1, NEUROD1, and synaptophysin (SYP), SCCC is classified into an inflammatory phenotype (SCCC-I) and a neuroendocrine phenotype (SCCC-A/N) [68]. These subtypes exhibit significant differences in gene expression, mutation profiles, immune cell infiltration, and prognosis, providing an important basis for personalized treatment. The SCCC-I subtype shows enrichment of genes related to interferon-α/β (IFN-α/β) signaling and the major histocompatibility complex class II (MHC II) complex pathway, upregulation of genes involved in inflammatory response and epithelial–mesenchymal transition (EMT), and suppression of proliferation and DNA repair pathway genes. This phenotype harbors more PIK3CA mutations, contains a higher number of T cells (including CD8^+^ T cells), and tends to show higher programmed death-ligand 1 (PD-L1) expression with a relatively greater degree of immune cell infiltration, characterized primarily by an “immune-inflamed” profile. Clinically, patients with this subtype have significantly higher survival rates and objective response rates compared to other subtypes and may benefit from combination therapy with immune checkpoint inhibitors (ICIs). The SCCC-A/N subtype highly expresses neuroendocrine markers (e.g., SYP, neural cell adhesion molecule 1 (NCAM1), insulinoma associated 1 (INSM1)) and glycolysis-related genes (e.g., lactate dehydrogenase A (LDHA), solute carrier family 2 member 1 (SLC2A1)/glucose transporter 1 (GLUT1), glyceraldehyde-3-phosphate dehydrogenase (GAPDH)). Tumors of this subtype exhibit less intratumoral immune cell infiltration, characterized by “immune-excluded” or “immune-desert” features, and are associated with a relatively poorer prognosis. Higher SYP expression and more frequent necrosis are observed, indicating a more pronounced neuroendocrine phenotype [93]. Within this group, SCCC-A highly expresses ASCL1 with downregulated Notch signaling, while SCCC-N highly expresses NEUROD1 and its target genes [68]. Basic research indicates that this subtype is sensitive to chemotherapy but prone to developing resistance. Therefore, clinical management requires intensified monitoring after chemotherapy and the exploration of drugs targeting the neuroendocrine phenotype (e.g., SOX2 inhibitors). Pan et al., using a combination of techniques, found that the neuroendocrine group harbored more *TP53* mutations, while the SCCC-I group had more PIK3CA mutations. This provides new insights into tumor heterogeneity and potential therapies for SCCC, although further research is needed to determine whether SCCC classification correlates with distinct therapeutic vulnerabilities.

### 4.3. Exploration of Targeted Pathway Inhibitors and Immunotherapy Applications Based on Molecular Mechanisms

A clear consensus on the standard treatment regimen for small cell carcinoma of the cervix has not yet been reached [14]. Therefore, the search for novel biomarkers for targeted therapy and immunotherapy to improve patient outcomes is particularly crucial. Limited clinical efficacy drives basic research into molecular targeting and immunotherapy. Immunotherapy has shown great potential in cervical cancer treatment [127,128], including for patients with small cell carcinoma of the cervix [129,130]. Mabuchi et al. proposed that alterations in pathway genes such as RAS-MAPK and PI3K-Akt-mTOR are core targets [76,131,132], and novel drugs targeting these pathways can guide clinical targeted therapy: for example, patients with PIK3CA mutations may consider mTOR inhibitors [95,133,134,135]; patients with KRAS G12V mutations may be sensitive to MEK inhibitors [136]; poly (ADP-ribose) polymerase (PARP) inhibitors may also be suitable for patients with breast cancer 1/2 (BRCA1/2) mutations, etc. Regarding immunotherapy, immune checkpoint inhibitors (ICIs) targeting the programmed death 1 (PD-1)/PD-L1 axis are effective against SCCC [137,138], but PD-1/PD-L1 inhibitors are effective only in a subset of patients with a low single-agent response rate, driving research into dual CTLA-4 and PD-1 blockade [130,139]. A phase II clinical trial evaluating the efficacy of cadonilimab (AK104), a bispecific antibody targeting PD-1 and cytotoxic T-lymphocyte-associated protein 4 (CTLA-4), in recurrent SCCC is underway. Furthermore, potential targets discovered in basic research, such as the TFF3-ELF3 axis, OKT1, somatostatin receptor 1/2 (SSTR1/2), and SOX2; corresponding targeted strategies (such as selinexor downregulating SOX2) have been validated in other tumors [140,141], providing new directions for SCCC clinical treatment. Heat shock protein 90 (HSP90) inhibitors and HIF-1α targeting strategies may radiosensitize tumors [142], and the exploration of novel targets such as cell division cycle 20 (CDC20) and flap structure-specific endonuclease 1 (FEN1) and their corresponding therapeutic strategies all reflect the continuous empowerment of clinical treatment by basic research [111,142], forming a bidirectional cycle.

### 4.4. Drug Resistance, Recurrence, and Prognosis: Synergistic Regulation of Molecular Mechanisms and Clinical Management

In the disease course of small cell carcinoma of the cervix (SCCC), drug resistance, recurrence, and prognosis are closely interconnected, forming a complex and mutually influential pathological system. Among these, drug resistance is a key factor leading to disease recurrence and poor prognosis, operating like a hidden mastermind manipulating the trajectory of disease progression. Delving into the mechanisms of drug resistance in SCCC is not only crucial for understanding the nature of the disease but also forms an essential foundation for developing effective treatment strategies and improving patient prognosis.

#### 4.4.1. Decoding Drug Resistance: The Molecular Logic Behind Therapeutic Dilemmas

The intrinsic biological characteristics of SCCC cells constitute a critical basis for their drug resistance, which is a fundamental cause of the high recurrence rate and poor prognosis. The functional loss of tumor suppressor genes *RB1* and *TP53* disrupts DNA damage repair, rendering tumor cells unable to initiate apoptosis during radiotherapy or chemotherapy, thereby leading to primary resistance. Combining chemotherapy with DNA damage repair inhibitors can reduce the incidence of such resistance. Although chemotherapy remains a mainstay for SCCC, treatment failure frequently occurs due to tumor heterogeneity and therapeutic resistance. Chemotherapeutic agents can induce a phenotypic shift in SCCC cells towards an adenocarcinoma-like state, and enhanced drug metabolism mediated by the n-acetyltransferase 1 (NAT1) gene further exacerbates chemoresistance [143]. Remodeling of the tumor microenvironment also contributes to resistance. Infiltration of regulatory T (Treg) cells suppresses immune responses, enabling tumor cells to evade immune attack, while secreted cytokines promote tumor growth and drug resistance. Secretagogin (SCGN), a hexa-EF-hand calcium-binding protein that enhances insulin secretion, confers chemoresistance by regulating the cell cycle and apoptosis [144]. Aberrant activation of signaling pathways such as PI3K-Akt-mTOR and MAPK provides tumor cells with a survival advantage against therapeutic damage. These molecular mechanisms collectively limit the efficacy of SCCC treatments and lay the groundwork for subsequent recurrence.

#### 4.4.2. Dynamic Surveillance: Molecular Monitoring and Intervention for Recurrence Risk

Building upon the molecular foundations of therapy resistance, targeted clinical strategies for monitoring recurrence have been established. However, due to the highly aggressive nature of SCCC and its characteristic early, high recurrence rate, constant vigilance for the presence of distant micrometastases is required [144,145]. Recurrence can occur both locally and as distant metastasis [146], with the latter associated with a poorer prognosis and greater therapeutic difficulty. Clinical guidelines recommend follow-up examinations every 3 to 6 months for the first 2 years post-treatment, every 6 to 12 months for the subsequent 3 years, and lifelong surveillance thereafter, with evaluation at any time suspicious symptoms arise. Follow-up assessments encompass pelvic examination, serum NSE testing, and imaging studies. Biopsy confirmation is recommended whenever recurrence is suspected. For patients who have received radiotherapy, regular brain MRI surveillance is advised to monitor for central nervous system recurrence. These clinical decisions are informed by the molecular understanding that “drug-resistant tumor cells are prone to forming distant micrometastases”. Concurrently, novel monitoring technologies emerging from basic research are gradually translating into clinical practice. Circulating tumor DNA (ctDNA) assessment offers a non-invasive advantage for the dynamic monitoring of tumor burden [147,148], leading to the clinical recommendation for ctDNA methylation testing at 3–6-month intervals. Furthermore, ultra-low-pass whole-genome sequencing (ULP-WGS), being cost-effective [149,150], can evaluate genome-wide tumor burden and resistance profiles. Although current data in SCCC are limited [151], it provides a potential avenue for early warning of precise recurrence. A high tumor fraction at diagnosis or upon recurrence, along with additional copy number gains in genes of pathways such as PI3K/Akt [151], suggests distant visceral metastasis and a high disease burden, which is consistent with the mechanistic insight from basic research that “aberrant pathway activation enhances tumor invasiveness”.

#### 4.4.3. Prognostic Model Reconstruction: Building an Integrated Predictive System Combining Molecular and Clinical Factors

The prognosis assessment of small cell carcinoma of the cervix (SCCC) is a complex process that requires the comprehensive consideration of multiple factors. Deeply integrating molecular biomarkers with clinical factors enables a transition from empirical judgment to precise prediction, providing more scientific and accurate guidance for patient treatment and management.

The most extensively researched and core prognostic category for SCCC encompasses clinicopathological features, which are essentially the external manifestations of accumulated molecular abnormalities within the tumor. Advanced FIGO stage [9,12,29,73,152,153,154,155], lymph node metastasis and its ratio [71,120,156,157,158,159], and tumor diameter > 4 cm are directly linked to tumor burden and metastatic risk [13], serving as core indicators of poor prognosis. Positive surgical margins and LVSI positivity are associated with an increased risk of recurrence due to residual disease, necessitating intensified postoperative surveillance. Additionally, deep stromal invasion, pure small cell histology, age over 60 years, and smoking history are indicative of a worse prognosis. Conversely, patients with shallow stromal invasion, young age (<45 years), or a polypoid growth pattern (which may limit tumor spread) generally have a more favorable prognosis [13,14,74,153,160,161]. Treatment-related factors are equally crucial [57]. For early-stage patients, radical hysterectomy combined with lymphadenectomy is associated with a better prognosis compared to radiotherapy alone; surgery primarily benefits patients with a low risk of recurrence, such as those with negative lymph nodes and FIGO stage I disease [162]. Concurrent or adjuvant chemotherapy can also improve survival [120,163], with an adequate number of chemotherapy cycles being key to efficacy [63]. However, the prognostic benefit of neoadjuvant chemotherapy remains controversial [29,57]. These clinical factors are interwoven, collectively influencing patient outcomes, and clinicians must consider them comprehensively to formulate individualized treatment plans.

At the molecular and biomarker level, basic research has confirmed that markers such as CgA positivity [29], Syn negativity, CD56 negativity, and a high Ki-67 index are associated with enhanced tumor proliferation and invasive capacity [164], indicating a poor prognosis [165]. Overexpression of SOX2, alterations in the *RB1* gene, high expression of ERBB4, and specific expression of clonal genome rearrangement (CGR) contribute to increased genomic instability and are linked to reduced patient survival rates. Furthermore, the SCCC-I (immune-desert) subtype and a lack of CD8-positive T-cell inflammatory infiltration also predict a poor prognosis [140,164,166,167]. To achieve more accurate prognostic prediction, a new trend has emerged: integrating molecular biomarkers with clinical factors into prognostic scoring models. These “molecular–clinical integrated models” fully incorporate information from both molecular and clinical dimensions, enabling a more comprehensive and accurate assessment of patient prognosis.

## 5. Discussion

Current research on small cell carcinoma of the cervix (SCCC) remains confronted with dual bottlenecks in both basic mechanisms and clinical translation. At the foundational level, the carcinogenic drivers of HPV-negative subtypes have yet to be clearly identified, and existing research models each have limitations: CTOS cell lines lack microenvironmental interactions; organoid models, while capable of simulating three-dimensional structures, are difficult to popularize due to their complex construction; and genetically engineered mouse and PDX models suffer from long cycles and high costs. In clinical translation, prominent challenges include the scarcity of novel targeted drugs, insufficient accuracy in early diagnosis, limited specificity of biomarkers, and further complications arising from debates over treatment resistance and clinical decision-making regarding neoadjuvant chemotherapy.

Breaking through these bottlenecks depends on constructing a closed-loop research system that starts with clinical questions, is driven by technological innovation, and aims to achieve precision diagnosis and treatment. Future efforts must focus on the interaction mechanisms between the tumor microenvironment and differentiation regulation, strive to deepen the analysis of dual-track carcinogenic mechanisms—both HPV-dependent and HPV-independent—leverage CRISPR gene editing to construct precise mutation models, and combine quantitative proteomics with single-cell sequencing to uncover new therapeutic targets. Model development should promote the standardization and accessibility of cutting-edge technologies such as organoids and gene-edited models, balancing simulation fidelity with practical feasibility [168]. Clinical translation requires the implementation of subtype-specific strategies and conducting prospective clinical trials of targeted and immunotherapies based on molecular subtyping [169]. Simultaneously, prevention systems should be built on the dominant role of the HPV18 subtype, verifying the long-term preventive efficacy of the nine-valent vaccine and exploring the chemopreventive value of HPV E6/E7 inhibitors for high-risk populations. In summary, the future core lies in multidisciplinary integration: standardizing research systems through the establishment of international multicenter biobanks, improving the efficiency of pathological subtyping with artificial intelligence, and promoting the clinical validation of sequential “chemotherapy–targeted therapy–immunotherapy” strategies [93]. Only through deep reciprocal feedback and dynamic iteration between basic research and clinical practice can we truly overcome the current limitations in SCCC diagnosis and treatment, guide it from an unmanageable predicament onto a track of precise regulation, and fundamentally realize the leap from understanding to therapy.

## Data Availability

No new data were created or analyzed in this study. Data sharing is not applicable to this article.

## Abbreviations

The following abbreviations are used in this manuscript:

Abbreviations	Meaning
SCCC	small cell carcinoma of the cervix
CC	cervical cancer
CSCC	cervical squamous cell carcinoma
CA	cervical adenocarcinoma
hr-HPV	high-risk human papillomavirus
TP53	tumor protein 53
RB1	retinoblastoma 1
CgA	chromogranin A
Syn	synaptophysin
CD56	neural cell adhesion molecule
NSE	neuron-specific enolase
INSM1	insulinoma-associated protein 1
SCGN	secretagogin
IHC	immunohistochemistry
ISH	in situ hybridization
PCR	polymerase chain reaction
LVSI	lymphovascular space invasion
LNM	lymph node metastasis
NCCN	National Comprehensive Cancer Network
SCLC	small cell lung cancer
SGO	Society of Gynecologic Oncology
GCIG	Gynecologic Cancer InterGroup
DFS	disease-free survival
LCR	long control region
URR	upstream regulatory region
pRb	retinoblastoma protein
CDKs	cyclin-dependent kinases
mRNA	messenger ribonucleic acid
SOX2	SRY-box transcription factor 2
MYC	MYC proto-oncogene family
NR4A	nuclear receptor subfamily 4 group A family
ANKRD	ankyrin repeat domain-containing family
FGFR3-TACC3	fibroblast growth factor receptor 3-transforming acidic coiled-coil containing protein 3
ANKRD12-NDUFV2	ankyrin repeat domain-containing protein 12-NADH dehydrogenase flavoprotein 2
PI3K	phosphatidylinositol 3-kinase
Akt	protein Kinase B
TCGA	The Cancer Genome Atlas
CNVs	copy number variations
PIK3CA	phosphatidylinositol-4,5-bisphosphate 3-kinase catalytic subunit alpha
KRAS	KRAS proto-oncogene, GTPase
PTEN	phosphatase and tensin homolog
FAK	focal adhesion kinase
JNK	c-Jun N-terminal kinase
RAF	rapidly accelerated fibrosarcoma
ERK	extracellular regulated protein kinase
MAPK	mitogen-activated protein kinase
MEK	mitogen-activated protein kinase kinase
ERBB4	erb-b2 receptor tyrosine kinase 4
ATRX	alpha thalassemia/mental retardation syndrome X-linked
FGFRL1	fibroblast growth factor receptor-like 1
STK39	serine/threonine kinase 39
HIF-1α	hypoxia-inducible factor-1α
VEGF	vascular endothelial growth factor
MMP-2	matrix metalloproteinase-2
MMP-9	matrix metalloproteinase-9
ASCL1	achaete-scute family bHLH transcription factor 1
NEUROD1	neuronal differentiation 1
SLFN11	schlafen family member 11
POU2F3	POU class 2 homeobox 3
YAP1	yes-associated protein 1
PARP	poly (ADP-ribose) polymerase
mTOR	mammalian target of rapamycin
CD8^+^	cluster of differentiation 8
SPP1^+^	secreted phosphoprotein 1
PDGF	platelet-derived growth factor
HDAC	histone deacetylase
dMMR	deficient mismatch repair
SYP	synaptophysin
CD74	cluster of differentiation 74
IFN-α/β	interferon-α/β
EMT	epithelial–mesenchymal transition
PD-L1	programmed death-ligand 1
ICIs	immune checkpoint inhibitors
NCAM1	neural cell adhesion molecule 1
INSM1	insulinoma associated 1
LDHA	lactate dehydrogenase A
GLUT1	solute carrier family 2 member 1 (SLC2A1)/glucose transporter 1
GAPDH	glyceraldehyde-3-phosphate dehydrogenase
BRCA1/2	breast cancer 1/2
PD-1	programmed death 1
CTLA-4	cytotoxic T-lymphocyte associated protein 4
SSTR1/2	somatostatin receptor 1/2
HSP90	heat shock protein 90
CDC20	cell division cycle 20
FEN1	flap structure-specific endonuclease 1
NAT1	N-acetyltransferase 1
Treg	regulatory T
ctDNA	circulating tumor DNA
ULP-WGS	ultra-low-pass whole-genome sequencing
CGR	clonal genome rearrangement
